# Prevalence of Agenesis of Mandibular Third Molars in A Tertiary Healthcare Centre of Nepal

**DOI:** 10.31729/jnma.4421

**Published:** 2019-06-30

**Authors:** Pragya Regmee, Jyotsna Rimal, Incha Kumar Maharjan, Surya Raj Niraula

**Affiliations:** 1 Department of Oral Medicine and Radiology, B. P. Koirala Institute of Health Sciences, Dharan, Nepal; 2 School of Public Health and Commmunity Medicine, B.P. Koirala Institute of Health Sciences, Dharan, Nepal

**Keywords:** *anodontia*, *mandible*, *molar*, *Nepal*, *panoramic radiography*

## Abstract

**Introduction:**

Racial variation, genetic inheritance and various other factors can affect the jaw size and ultimately the tooth size and number. Studies for agenesis of mandibular third molars have been carried out in various populations but the data relating to these are not evident from most of the parts of Nepal. Hence, the objective of the present study is to determine the prevalence of agenesis of mandibular third molars among the population of patients visiting the Department of Oral Medicine and Radiology of B. P. Koirala Institute of Health Sciences.

**Methods:**

This descriptive cross-sectional study was conducted at Department of Oral Medicine And Radiology, College of Dental Surgery, B. P. Koirala Institute of Health Sciences from September 2015 to September 2016 after taking ethical approval from Institutional Review Committee. Two hundred and eighty four patients (568 sites of third molar bilaterally), visiting the Department of Oral Medicine and Radiology were studied with Panoramic Radiograph to assess for agenesis of mandibular third molars bilaterally. Data was entered in Microsoft Excel sheet and transferred to Statistical Package for the Social Sciences version 11.5.

**Results:**

Out of 284 participants, 568 sites of mandibular third molars were evaluated and agenesis was seen among 163 (28.7%) participants at the confidence interval of 95% (28.643 to 28.757). Total numbers of patient with single missing mandibular third molar were 35 (6.2%). Twenty one had agenesis only on the right side and 14 had agenesis only on the left. The total number of patients with agenesis of both the mandibular third molars was 64 (22.5%).

**Conclusions:**

Agenesis was highly prevalent in this study group. The likelihood of third molar being absent on one side, when there was concurrent missing third molar on the other side of mandible was also high.

## INTRODUCTION

A wisdom tooth or third molar (M3) is one of the three molars per quadrant of the human dentition. It is the most posterior of the three molar teeth. M3 generally erupts between the ages of 17 and 21 years.^1^ Tooth agenesis is the most common developmental anomaly of human dentition, occurring most often in the third molars.^2^

There are various factors that affect the jaw size and ultimatelythetooth size and number.^3–5^ Hence, the studies of prevalence and incidence of agenesis of third molars have been carried out on different population groups by various authors.^6–8^ Unfortunately, data relating to these are available from only negligible reports from Nepal.

Thus, the objective of this study is to determine the prevalence of agenesis of mandibular third molar in patients visiting the Department of Oral Medicine and Radiology of B. P. Koirala Institute of Health Sciences.

## METHODS

This was a descriptive cross-sectional study, done on patients indicated for panoramic radiograph. It was conducted in the Department of Oral Medicine and Radiology, College of Dental Surgery, B. P. Koirala Institute of Health Sciences (BPKIHS). The duration of study was one year from September 2015 to September 2016.

This study was conducted after the approval from Institutional Review Committee of BPKIHS (Ref No: IRC/511/015). Information sheet was provided to all the participants of the study and written informed consent was taken.

Patients, who were above 17 years, had an indication for panoramic radiograph and who were willing to give consent were included in the study. Patient with history of extraction of permanent molars, patients with developmental anomaly of face, congenital or systemic disease and/or major pathology in the mandible that has/had caused severe bone resorption/destruction, ankylosis, facial asymmetry, bone expansion, root resorption and tooth migration, patients with multiple impacted or multiple supernumerary teeth and patients not willing to give consent were excluded from the study.

The sample size was calculated using the following formula,


$$\text{n}={\text{Z}}^{2}\times (\text{p}\times \text{q})/{\text{d}}^{2}$$

where,

n = sample size
p = prevalence, 9%^3^
q = 1-p
d = margin of error, 4%
Z = 1.96 at 95% CI

Sample size was calculated was 196. With the non-response rate of 10%, the sample size was found to be 215, however, the study was done among 284 patients. Convenient sampling was done.

The included samples were categorized according to gender and also according to age as follows: Category F (First group corresponding to the age group 17 to 26 years), Category S (Second group corresponding to the age group 27 to 36 years) and Category T (Third group corresponding to the age group 37 years and above).

History was taken and patients were examined clinically under aseptic condition. Radiographs were taken by Panoramic Machine (GendexOrthoralix 9200 DDE). Images were produced by digital imaging technique.

A predesigned proforma was filled for each patient volunteering in the study. Confidentiality and privacy of the patients were maintained. Data was entered in Microsoft Excel sheet and transferred to Statistical Package for the Social Sciences (SPSS) version 11.5.

Amongst all the samples the percentage of third molar agenesis were calculated in following manner: total number of M3 missing in total sites examined (percentage) and then percentage of patients with single missing mandibular third molar and percentage of patients with both the mandibular third molars being absent, without categorizing into any further groups, but according to gender.

## RESULTS

The total number of M3 missing were 163 (28.7%) at the confidence interval of 95% (28.643 to 28.757) out of the total 568 sites evaluated. Total number of patient with single missing mandibular third molar were 35 (21 had agenesis only on the right side and 14 had agenesis only on the left). The percentage for which came out to be 6.2%. The total number of patients with agenesis of both the mandibular third molars was 64 (22.5%) (Table 1).

**Table 1.** Agenesis on different sides.

Mandibular third molar	On right side n (%)	On left side n (%)	On both sides n (%)
Agenesis	85 (29.9)	78 (27.5)	64 (22.5)
Present	199 (70.1)	206 (72.5)	185 (65.14)
Total	284 (100)	284 (100)	249 (100)

A total of 284 patients, that is, 568 sites of mandibular third molars bilaterally were evaluated. The mean± SD age of participants was 30.10± 10.22 years.

Total number of female participants were 156 (54.9%) and the remaining 128 (45.1%) were male. In female, 46 (29.5%) M3 were missing on the right side and 44 (28.2%) M3 were missing on the left side. In male, 39 (30.5%) M3 were missing on the right side while 34 (26.6%) were missing on the left side. Calculating the overall agenesis, it was almost similar in male and female (Table 2).

**Table 2.** Gender and sidewise distribution of agenesis.

		Right side		Left side	
Gender	Total	Agenesis n (%)	Present n (%)	Agenesis n (%)	Present n (%)
Female	156	46 (29.5)	110 (70.5)	44 (28.2)	112 (71.8)
Male	128	39 (30.5)	89 (69.5)	34 (26.6)	94 (73.4)
Total	284	85 (29.9)	199 (70.1)	78 (27.5)	206 (72.5)

T on both sides (33 on the right side and 34 on the left side) (Table 3).

**Table 3.** Age category and sidewise distribution of agenesis

Age category	Number of observation	Right side Agenesis	Present (%)	Left side Agenesis (%)	Present (%)
F	140	23 (16.4 %)	117 (83.6 %)	17 (12.1 %)	123 (87.9 %)
S	81	29 (35.8 %)	52 (64.2 %)	27 (33.3 %)	54 (33.3 %)
T	63	33 (52.4 %)	30 (47.6 %)	34 (54.0 %)	29 (46%)
Total	284	85 (29.9 %)	199 (70.1 %)	78 (27.5 %)	206 (73%)

## DISCUSSION

Mandibular third molar agenesis is often a frequent dental discovery these days. This finding is sometimes detected intentionally while at most of the times it remains unnoticed until patient undergoes radiographic investigations for other dental problems. Numerous studies have been conducted from across the globe to find the prevalence of agenesis of mandibular third molar.^9^

While assessing agenesis in this study, total mandibular third molar (M3) missing were 28.7%. This finding was almost similar to the finding in the study by Sapoka and Demirjian, done in French Canadian dental patients in 1971, where the percentage of mandibular third molar agenesis was 25.4%.^10^ Also, another study conducted by Harris and Clark in American blacks and whites showed the prevalence to be close to the current study (27%) but this was the prevalence of overall missing teeth rather than third molar per se.^11^ Hence, the finding in this study seems that it is the highest percentage of prevalence of agenesis of mandibular third molar.

In contrary, the study by Barka et al, Kazanci et al, and Marzola et al. showed the prevalence of missing mandibular third molar to be 15.5%, 10.35%, and 3.52% respectively.^12–14^ In the study conducted by Upadhyaya et al. in Dhulikhel, the prevalence of missing 38 was 8.84%, missing 48 was 11.56% and missing 38 and 48 together were seen in 8.16%.^15^ This difference from other studies could be attributed to the differences in sample sizes besides the racial, topographic and anthropometric differences. The systematic review and metaanalysis by Carter and Worthington also helps in explaining this finding that there is clear significant differences among many of the populations for agenesis.^8^

In the present study, total percentage of patients with single missing mandibular third molar were 6.2% and those with both the mandibular third molar being absent were 22.5%. This finding was consistent with the findings in studies by Barka et al. and Mani et al, which states that bilateral agenesis is more common than unilateral agenesis.^12,16^ In the study conducted by Mani et al, bilaterally missing mandibular third molar was seen in 7.2% of children and unilaterally missing mandibular third molar was seen in 5.8% of the children.^16^ In the study conducted by Barka et al, bilaterally missing mandibular third molar was seen in 12.1% and unilaterally missing mandibular third molar was seen in 6.8%.^12^

Also, in the present study the difference in gender wise distribution of agenesis was statistically insignificant between female and male (28.8% in female and 28.5% in male). This finding is higher in percentage as compared to the study conducted by Rozkovcove (7% in female and 7.5% in male).^17^ Also, no significant difference in third molar agenesis was noted between gender in the study by Kazanci et al, Celikoglu et al. and Mani et al.^13,16,18^

However, in the study conducted by Harris and Clark; and Byahatti and Ingafou significant sex differences were found for the third molar agenesis (absence more common in females),^11,19^ and the gender differences were greater in whites than in blacks.^11^ In the study conducted by Upadhyaya et al, agenesis was more prevalent in males (67.02%) than in females (42.5%).^15^

A significant difference could be found in this study on agenesis with respect to different age groups. Maximum number of absent M3 was found in the age group of more than 37 years and minimum was found in the age group of 17 to 26 yrs. The reason for this difference may be because of the inconsistently chosen or non-randomized population sample. But, this finding challenges the current concept that evolution and change in dietary habits are responsible factors for teeth agenesis.

These differences in agenesis in different gender or age groups could also be because of the fact that agenesis appears to be an inherited characteristic.^20^ And as stated by Haga et al, genetic basis exists for third molar agenesis and this genetic basis also explains why agenesis is more prevalent in one race or in one age group or in one gender but not in the others.^2^ The statement by De Coster also tries to explain this difference, which states that there are different genes involved in human non-syndromichypodontia that play critical roles during early craniofacial development. But even though researches are going on regarding the different factors responsible for tooth agenesis, till date a definitive factor has not been established and tooth agenesis still appears to be multifactorial.^21^

While analyzing the association of M3 agenesis on the right and left side, this study has shown that those who had agenesis on one side had more likelihood to have agenesis on the other side as well. There are many literatures which have shown higher chance of missing M3 on both the sides of mandible as compared to one side.^12,15–17^ In the study by Mani et al, the odds of any 3^rd^ molar missing were increased 3.3 times when there was any other missing tooth.^16^ Likewise, studies conducted by Hirakata et al. and Sanpei et al. has shown association between agenesis of any other teeth when there was presence of bilateral agenesis of mandibular or maxillary third molars.^22,23^

Regarding the limitation of the study, one major concern with this study is the lack of random sampling, which was avoided to prevent unnecessary radiographic exposure to the patients.

## CONCLUSIONS

From this study we could conclude that, mandibular third molar agenesis was highly prevalent in this study population. The likelihood of M3 being absent on one side, when there was concurrent missing M3 on the other side of mandible was also high. Difference in agenesis with respect to different age category was also found to be statistically significant.

For future scope of similar kind of studies, we recommend genetic studies to understand the genetic basis of agenesis of mandibular third molar or any other teeth. Studies with larger sample size can be conducted in different parts of the country. This will generate national database of exact prevalence of agenesis of M3 and will ultimately contribute to the knowledge regarding dentitional evolution of humans as a whole.

## Conflict of Interest

**None.**
